# Nanoporous Organic Polymer/Cage Composite Membranes**

**DOI:** 10.1002/anie.201206339

**Published:** 2012-12-06

**Authors:** Alexandra F Bushell, Peter M Budd, Martin P Attfield, James T A Jones, Tom Hasell, Andrew I Cooper, Paola Bernardo, Fabio Bazzarelli, Gabriele Clarizia, Johannes C Jansen

**Affiliations:** School of Chemistry, University of ManchesterOxford Road, Manchester M13 9PL (UK) E-mail: peter.budd@manchester.ac.uk; Department of Chemistry, University of LiverpoolCrown Street, Liverpool L69 7ZD (UK) E-mail: aicooper@liverpool.ac.uk; Institute on Membrane TechnologyITM-CNR, Via P. Bucci 17/C, 87030 Rende (CS) (Italy) E-mail: johannescarolus.jansen@cnr.it

**Keywords:** gas separation, membranes, microporous materials, organic–organic composites, polymers

There is an urgent need to develop efficient and economic CO_2_ purification technologies to upgrade waste CO_2_ to a reusable purity. Membrane-based separation processes are seen as one of the possible solutions to this problem.^1^ For large-volume membrane applications, such as CO_2_ recovery, high permeability is essential to minimize the membrane area, in combination with good selectivity.

For membrane applications, high free-volume polymers^2^ exhibit good processability, but they are prone to physical ageing. As transport depends on free volume, physical ageing leads to loss of permeability over time.^3^ Porous crystalline solids can give good transport properties, but are less easily fabricated into mechanically stable membranes. Combinations of polymers with inorganic or metal–organic particles in composite or mixed-matrix membranes (MMMs)^4^ may give synergistic enhancements in performance, but difficulties are encountered in achieving good dispersion within the membrane.^5^ Largely unexplored is the potential of purely organic dispersed phases, comprising only C, H, N, and O atoms, which should show better compatibility with a continuous polymeric matrix and which offer scope for tailoring the physical properties through organic synthesis.

Herein we demonstrate a novel route to MMMs in which the dispersed phase is generated by in situ crystallization of porous organic cage molecules from a single homogeneous, molecular solution. The incorporation of porous organic cages significantly enhances permeability, whereas chemically reduced, nonporous cage molecules have an opposite effect. We also compare the gas separation performance of membranes where crystals were generated by in situ crystallization against membranes where pre-formed nanocrystals were dispersed by co-casting with the polymer.

The crystallizable precursor is CC3 (Figure 1 a), which has approximately triangular windows of effective diameter 0.6 nm, which is large enough to admit gases and small organic molecules.^6^ The imine-linked CC3 was prepared as a powder with a Brunauer–Emmett–Teller (BET) surface area of 620 m^2^ g^−1^, based on N_2_ adsorption at 77 K. A suspension of racemic CC3 nanocrystals (nanoCC3) in dichloromethane was also prepared. The isolated nanocrystalline CC3 had a BET surface area of 770 m^2^ g^−1^. To examine the importance of rigidity and shape persistence in CC3, its reduced amine form was prepared. Complete reduction with sodium borohydride of all 12 imine linkages in CC3 results in transformation to a much less rigid dodecaamine molecule (redCC3), which does not exhibit permanent porosity in the solid state and which is amorphous in powder form.

**Figure 1:**
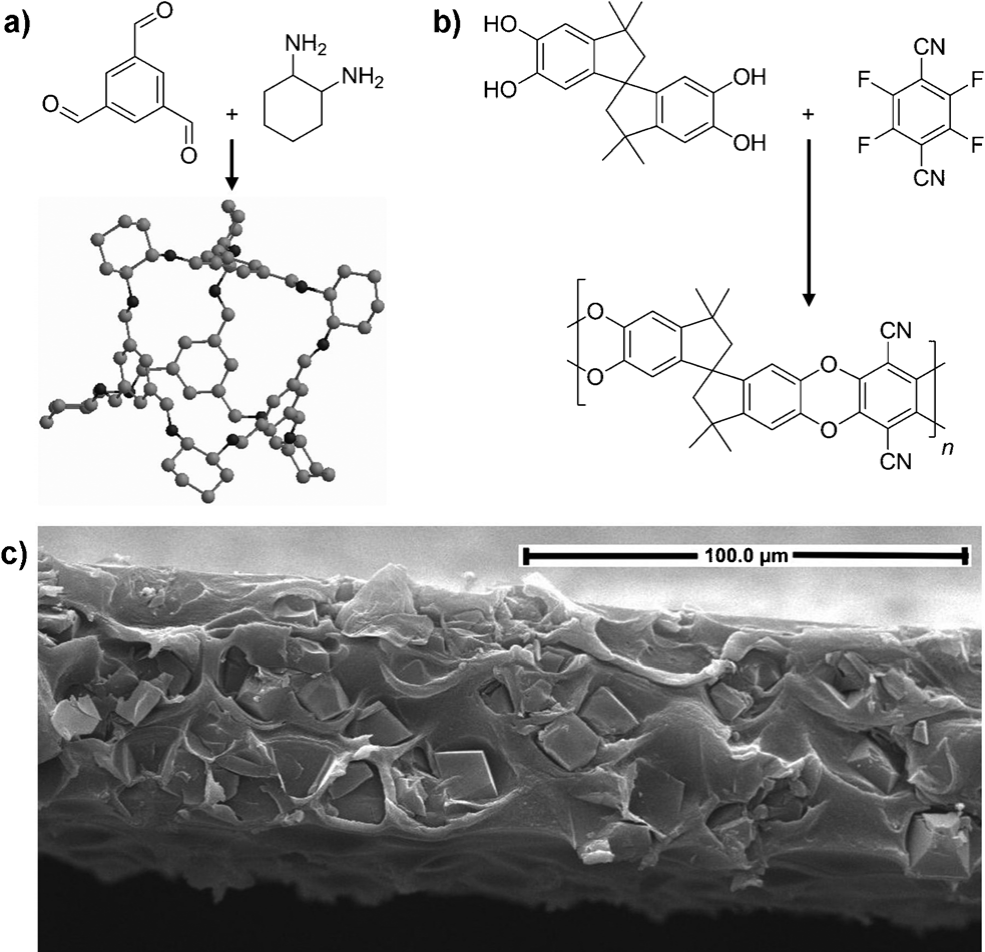
a) Porous imine cage CC3 synthesized from 1,3,5-triformylbenzene and (*R*,*R*)-1,2-diaminocyclohexane by a condensation reaction. b) PIM-1 is synthesized from 5,5′,6,6′-tetrahydroxy-3,3,3′,3′-tetramethyl-1,1′-spirobisindane and 1,4-dicyanotetrafluorobenzene by a step polymerization involving a double aromatic nucleophilic substitution. c) SEM image of a cross-section of a PIM-1/CC3 composite membrane (weight ratio 10:2).

The polymeric matrix is a polymer of intrinsic microporosity, referred to as PIM-1 (Figure 1 b),^7^ which exhibits membrane gas separation behavior at the current upper bound of performance^8^ for important gas pairs, such as CO_2_/N_2_. In the solid state, PIM-1 is an amorphous glassy polymer with a BET surface area of 770 m^2^ g^−1^, which is comparable to that of CC3. Both crystalline CC3 and amorphous PIM-1 gave N_2_ sorption isotherms that exhibit high uptake at very low relative pressure, indicative of a microporous material as defined by IUPAC (pore size <2 nm)^9^ (Supporting Information, Figure S7).

MMMs of PIM-1 with CC3 and redCC3 were prepared from homogeneous molecular solutions of the polymer and the cage molecule in CHCl_3_ by slow solvent evaporation, with polymer/cage weight ratios of 10:1, 10:2, and 10:3, corresponding to cage weight fractions of 0.09, 0.17, and 0.23. MMMs were also prepared from PIM-1 with preformed nanoCC3 in dichloromethane at weight ratios of 10:2 and 10:3. It should be noted that the preformed nanoCC3 powder does not, unlike CC3, dissolve in CH_2_Cl_2_ because it is prepared as a racemate that is very much less soluble^10^ than the homochiral CC3 molecule used to prepare MMMs by in situ crystallization.

For PIM-1/CC3 MMMs, scanning electron microscopy (SEM) shows crystals of dimensions 5–10 μm embedded within the membrane (Figure 1 c). The presence of crystalline CC3 was confirmed by X-ray diffraction (XRD). Area powder XRD studies demonstrated reasonable uniformity of CC3 distribution across the area of the membrane. For PIM-1/nanoCC3 MMMs, SEM showed a particulate structure with an average particle size about 90 nm. The X-ray diffraction pattern for PIM-1/nanoCC3 MMMs was similar to that observed for CC3 crystals generated in situ. For PIM-1/redCC3 MMMs, there was no evidence of crystallinity.

Single gas-permeation data were obtained for MMMs at various cage weight fractions (Supporting Information, Tables S1–S3). Permeability coefficients *P* and diffusion coefficients *D* were determined for N_2_, CH_4_, O_2_, He, and CO_2_ at 25 °C. These are effective values for the MMMs, averaged out over the polymeric and dispersed phases. In the simplest model of permeation, permeability is the product of a diffusion term and a solubility term. Apparent solubility coefficients *S* were calculated as *S*=*P*/*D*. The ideal selectivity for a pair of gases is the ratio of permeabilities, α(A/B)=*P*_A_/*P*_B_. It is well-established for PIM membranes that an alcohol treatment (immersion in methanol or ethanol overnight, followed by drying) helps flush out residual solvent and open up the PIM-1 structure, resulting in dramatic increases in permeability.7c Thus, measurements were made for MMMs both as prepared and after ethanol treatment. Results for ethanol-treated membranes are shown in Figure 2 for CO_2_ and N_2_. Plots for all the gases studied, for both as prepared and ethanol-treated membranes, are in the Supporting Information.

**Figure 2:**
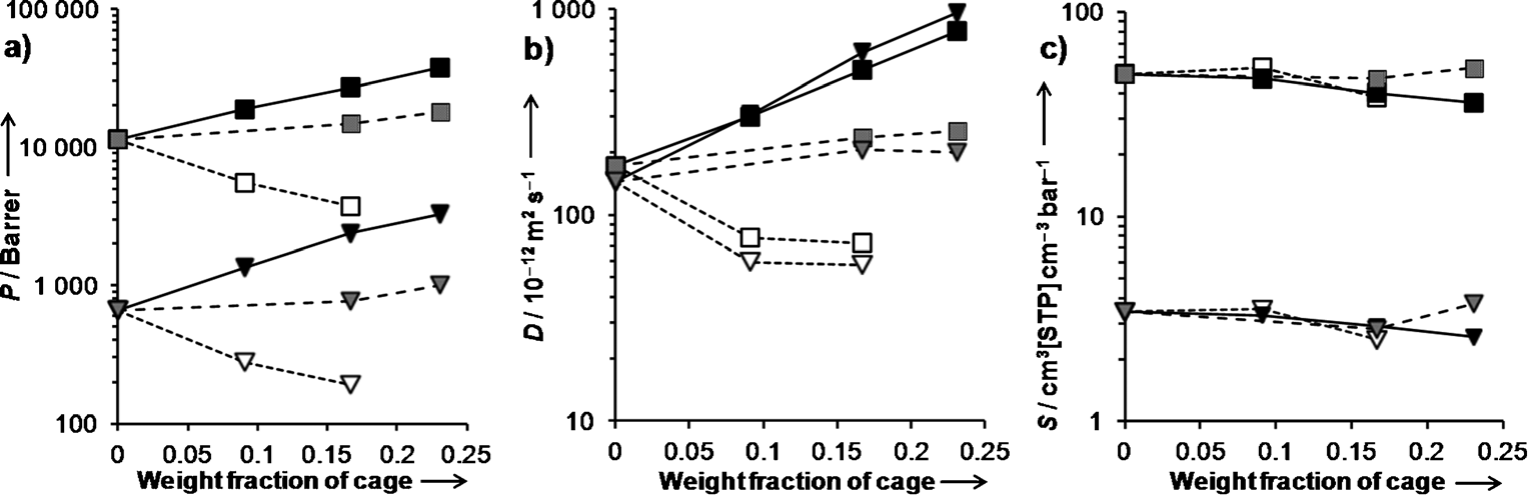
Dependence of a) permeability coefficient, b) diffusion coefficient, and c) solubility coefficient for CO_2_ (▪) and N_2_ (▾) on the weight fraction of cage for ethanol-treated PIM-1/CC3 (filled symbols, solid lines), PIM-1/redCC3 (open symbols, short dashes), and PIM-1/nanoCC3 (shaded symbols, long dashes). 1 Barrer=10^−10^ cm^3^[STP] cm cm^−2^ s^−1^ cmHg^−1^=3.35×10^−16^ mol m m^−2^ s^−1^ Pa^−1^.

Permeability increases with increasing weight fraction of CC3, but decreases with increasing weight fraction of redCC3. The changes in permeability reflect changes in diffusion coefficient, with intrinsically porous CC3 raising *D* and non-porous redCC3 lowering *D*. The enhanced diffusion with CC3 may be attributed to transport within the pore structure of crystalline CC3. Contributions associated with the interface between polymer and filler cannot be excluded, but such contributions are expected to be more evident in the case of nanofillers with a larger specific external surface, which was not observed. The reduced diffusion with redCC3 may be attributed to occupation of polymer free volume by molecularly dissolved redCC3. In contrast to the opposite effects of CC3 and redCC3 on *P* and *D*, both fillers lead to a slight reduction in solubility coefficients with increasing filler content. This reflects the dilution of PIM-1, which by itself exhibits extraordinarily high values of *S*.7c Selectivities relative to N_2_ slightly decrease with increasing CC3 content for CO_2_, He, and O_2_, but increase for CH_4_, while with redCC3 selectivities are approximately constant or slightly increase for all gases.

Comparing CC3 crystals generated in situ with pre-formed nanoCC3 for ethanol-treated membranes, the nanoCC3 has a much smaller effect on *D*, and hence *P*. The high values of *D* obtained with in situ crystallized CC3 suggest efficient transport through the relatively large crystals after ethanol treatment. In contrast, for as prepared membranes, values of *D* and *P* for nanoCC3 were similar to, or higher than, values for CC3, possibly because the more volatile and smaller solvent used with nanoCC3 (CH_2_Cl_2_ rather than CHCl_3_) left less residue. Values of *S* for ethanol-treated nanoCC3 membranes were comparable to, or higher than, those for the other fillers. The nanoCC3 membranes extend the upper bound of performance for various relevant gas pairs (Figure 3 a,b).

**Figure 3:**
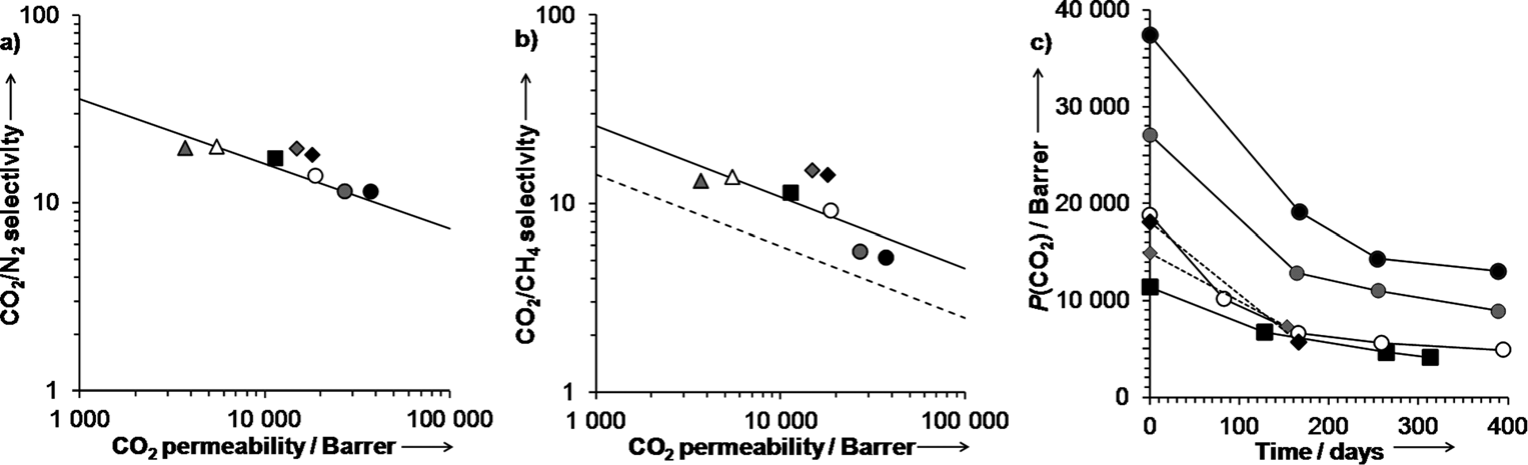
Double-logarithmic plots of selectivity versus permeability for a) CO_2_/N_2_ and b) CO_2_/CH_4_, showing Robeson’s 1991 (- - - -) and 2008 (—) upper bounds,^8^ and c) change in CO_2_ permeability over time. Experimental data for ethanol-treated PIM-1 (▪), and for PIM-1/CC3 MMMs (•), PIM-1/redCC3 MMMs (▴), and PIM-1/nanoCC3 MMMs (⧫) at weight ratios of 10:1 (open symbols), 10:2 (shaded symbols), and 10:3 (solid symbols).

Ethanol-treated PIM-1/CC3 (weight ratio 10:3) exhibits an extremely high CO_2_ permeability (37,400 Barrer), an order of magnitude higher than, for example, tetrazole-modified PIM-1,^11^ and within the range of values quoted for poly(1-trimethylsilyl-1-propyne) (PTMSP),^12^ which was long considered the most permeable polymer. However, amorphous, glassy polymers such as PTMSP lose excess free volume, and hence permeability, rapidly over time. Similarly, on ageing, ethanol-treated PIM-1 loses much of the extra permeability gained upon ethanol treatment. In contrast, the porosity within a crystalline filler should be stable, provided no chemical changes or irreversible adsorption occur. Figure 3 c shows changes in CO_2_ permeability with time after ethanol treatment for PIM-1/CC3 and PIM-1/nanoCC3 MMMs, and for a membrane of the same batch of PIM-1. With CC3 there is, as expected, some loss of permeability over time, reflecting PIM-1 as the dominant phase. Nevertheless, a significant increment is maintained when the crystalline CC3 is present. Thus, ethanol-treated PIM-1/CC3 (weight ratio 10:3) after more than one year still exhibits a CO_2_ permeability of 13 000 Barrer with a CO_2_/N_2_ ideal selectivity of 15. In contrast, the PIM-1/nanoCC3 at the highest loading loses all its additional permeability in less than six months, suggesting that transport enhancement is dominated by polymer bulk effects rather than by the porosity of the filler. The importance of the crystalline CC3 phase generated in situ is demonstrated by the fact that the sample with weight ratio of 10:3 has approaching three times the permeability of the sample with weight ratio of 10:1 after ageing. The rate of physical aging is much slower than that in the ultra-permeable PTMSP, which loses up to two orders of magnitude in permeability for oxygen and isobutane in 100 days.^13^

The performance with gas mixtures may differ from that of pure gases, particularly when strongly adsorbing species are present. Thus mixed gas permeation measurements were carried out using a synthetic ternary mixture (molar ratio CO_2_/O_2_/N_2_=35:10:55), which simulates the dry composition of typical flue gases from steel production or from lime kilns.^14^ For comparison, pure gas data were also obtained under similar conditions, using argon as a sweep gas. The results are presented in Figure 4. These permeabilities are lower than measured in the time lag mode, where the membrane is exposed to a much lower pressure, both in the feed side and in the permeate side. The CO_2_ permeability decreases with increasing (partial) pressure, both for the pure gas and for the gas mixture. This is due to the high solubility of CO_2_ in MMMs based on this type of polymer and highly porous fillers, leading to saturation of the Langmuir sorption sites, as predicted by the dual mode sorption mechanism. Pure nitrogen permeability is constant with pressure, whereas it decreases slightly in the mixed gas experiments. As a result, the selectivity of the membrane in mixed gases is superior to the ideal selectivity (Figure 4 b).

**Figure 4:**
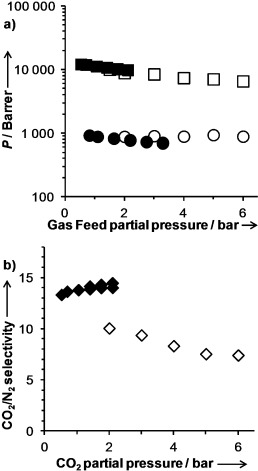
Pressure dependence of a) pure and mixed gas CO_2_ and N_2_ permeability and b) corresponding selectivity for ethanol-treated PIM-1/CC3 (weight ratio 10:3) aged for 258 days. CO_2_ permeability (▪), N_2_ permeability (•), and selectivity (⧫). Filled symbols represent the mixed gases, open symbols the pure gases.

In conclusion, these results demonstrate that the incorporation of a porous organic crystalline phase within PIM-1 can substantially enhance permeability while retaining good selectivity and providing better resistance towards physical ageing. The crystals generated in situ under the slow evaporation conditions used in this work are relatively large, but the size and distribution of the crystals can be modified by varying the processing conditions. In comparison to the use of dispersed preformed crystalline particles, the in situ crystallization route from a single homogeneous solution is a particularly convenient preparation method for MMMs. This approach is not limited to imine cage molecules, but should be readily extended to other functional “porous” organic molecules,^15^ including calixarenes, cucurbiturils, and other rigid, macrocyclic species.

## Experimental Section

Cage 3-R (CC3) was prepared using an improved method reported previously.6b Reduced cage 3-R (redCC3) was prepared from CC3 using a sodium borohydride reduction as described in the Supporting Information.

A suspension of nanocrystalline CC3 (nanoCC3) in CH_2_Cl_2_ was prepared as follows: CC3-*R* (1 g) was dissolved in CH_2_Cl_2_ (200 mL). An identical solution of the opposite enantiomer CC3-*S* was prepared. The *R* enantiomer solution, in a round-bottom flask, was chilled to −78 °C by immersion in an acetone/dry-ice bath. The *S* enantiomer solution was then added dropwise over 20 min with stirring at 300 rpm. Precipitation of nanocrystalline racemic CC3 occurs spontaneously upon mixing the two opposite chiral enantiomers.^10^

PIM-1 was synthesized by a step polymerization involving a double aromatic nucleophilic substitution from 5,5′,6,6′-tetrahydroxy-3,3,3′,3′-tetramethyl-1,1′-spirobisindane and 1,4-dicyanotetrafluorobenzene in dimethylformamide with K_2_CO_3_ at 65 °C, as described previously.7a

MMMs of PIM-1 with CC3, redCC3, and nanoCC3 were prepared as described in the Supporting Information. Materials and membranes were characterized by gas sorption analysis, scanning electron microscopy, and X-ray diffraction. Gas permeation tests of single gases were carried out at 25 °C and at a feed pressure of 1 bar, using a fixed-volume pressure increase instrument described elsewhere.^16^ Mixed gas permeation tests were carried out using an instrument equipped with a mass spectrometer.

## Supplementary material

Detailed facts of importance to specialist readers are published as ”Supporting Information”. Such documents are peer-reviewed, but not copy-edited or typeset. They are made available as submitted by the authors.
