# Supplementary material for: Nanoporous Organic Polymer/Cage Composite Membranes
Source: Angew Chem Int Ed Engl. 2012 Dec 6;52(4):1253–6. doi: 10.1002/anie.201206339 (PMC3734621; doi:10.1002/anie.201206339)
Supplement: Supplementary file 1 [file anie0052-1253-SD1.pdf]

Angewandte Chemie
Eine Zeitschrift der Gesellschaft Deutscher Chemiker

Supporting Information

© Wiley-VCH 2013

69451 Weinheim, Germany

Nanoporous Organic Polymer/Cage Composite Membranes**

Alexandra F. Bushell, Peter M. Budd, Martin P. Attfield, James T. A. Jones, Tom Hasell, Andrew I. Cooper,* Paola Bernardo, Fabio Bazzarelli, Gabriele Clarizia, and Johannes C. Jansen**

anie_201206339_sm_miscellaneous_information.pdf

CONTENTS

		page
1.	Preparation of redCC3	1
2.	Characterization of redCC3	1
3.	Preparation of mixed matrix membranes	3
4.	Visual appearance of mixed matrix membranes	3
5.	Gas sorption analysis	4
6.	Scanning Electron Microscopy (SEM)	5
7.	X-ray diffraction (XRD)	6
8.	Area powder X-ray diffraction	7
9.	Single gas permeation measurements	8
10.	Mixed gas permeation measurements	15

1. Preparation of redCC3

Reduced cage 3-R (redCC3) was prepared from CC3 using a sodium borohydride reduction as follows: 1.0 g (0.8944 mmol) CC3 was dissolved in dichloromethane (200 mL) at room temperature, after which methanol (150 mL) was added slowly, making sure the CC3 did not crash out of solution. With stirring, under nitrogen, sodium borohydride 440 mg (11.6 mmol) was added slowly (13 equivalents). The solution became cloudy and then cleared within 1 min. The solution was stirred for 24 h to ensure complete reduction. Once complete conversion had occurred, water (10 mL) was added, before removing the solvent under vacuum. The resulting white solid was placed in 100 mL $CHCl_3$ and 100 mL H_2O and placed in a separating funnel. The organic layer was retained, dried using $MgSO_4$ and filtered to give a clear, colourless solution. The chloroform was then removed under vacuum and the product obtained as a white solid (0.98 g, 95.9 % yield). ^{1}H NMR, 400 MHz (CDCl3) δ 7.10 (s, ArH, 12H), 3.82 (d, C*Hax*, 12H), 3.56 (d, CHeq, 12H), 2.2 (m, NC*Hax*, 12H), 2.0 (m, C*Heq*, 12H), 1.7 (m, C*Heq*, 12H), 1.1 ppm. (m, C*Hax*, 12H), 0.9 (m, C*Hax*, 12H) ^{13}C NMR (CDCl3) δ 141.3, 125.0, 61.3, 50.7, 31.7, 24.9 ppm. MALDI-TOF MS m/z: 1141.7 for $C_{72}H_{109}N_{12}$ [M] calculated for $C_{72}H_{109}N_{12}$ = 1141.702.

2. Characterization of redCC3

RedCC3 was characterized by ^{1}H NMR (**Figure S1**), ^{13}C NMR (**Figure S2**), HSQC NMR (**Figure S3**), COSY NMR (**Figure S4**) and Matrix-Assisted Laser Desorption/Ionization –Time of Flight (MALDI-TOF) mass spectrometry (**Figure S5**).

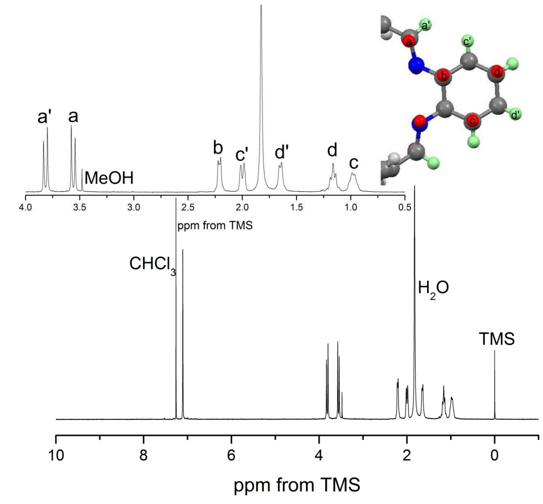

Figure S1. ^{1}H NMR spectra of reduced cage 3-*R* (redCC3). The red and green protons indicate those in axial and equatorial positions relative to the cyclohexyl ring.

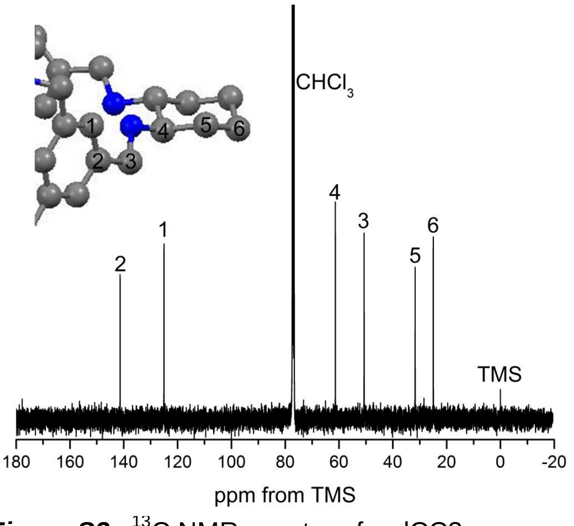

Figure S2. ^{13}C NMR spectra of redCC3.

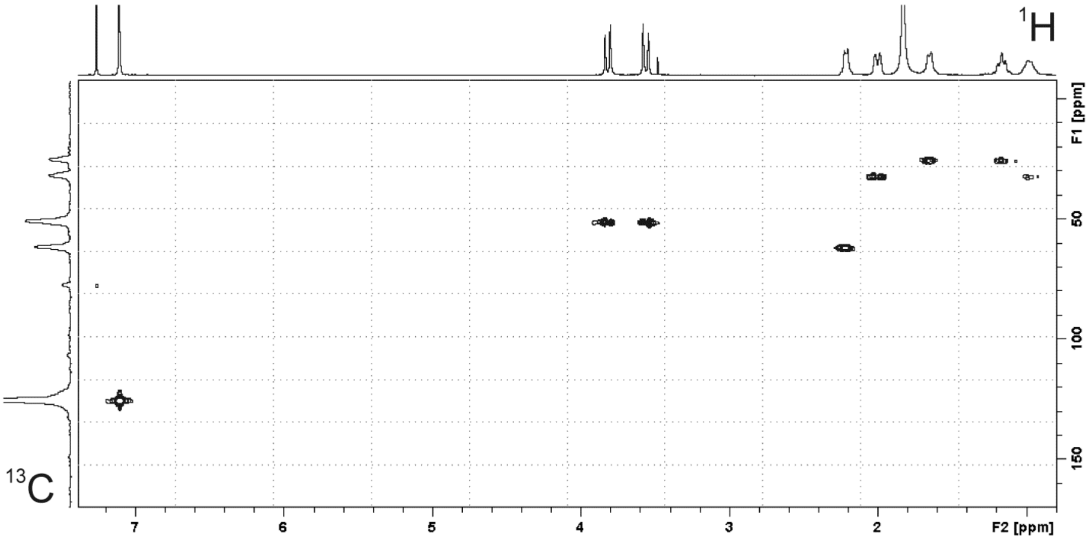

Figure S3. Heteronuclear Single Quantum Correlation (HSQC) NMR spectra of redCC3.

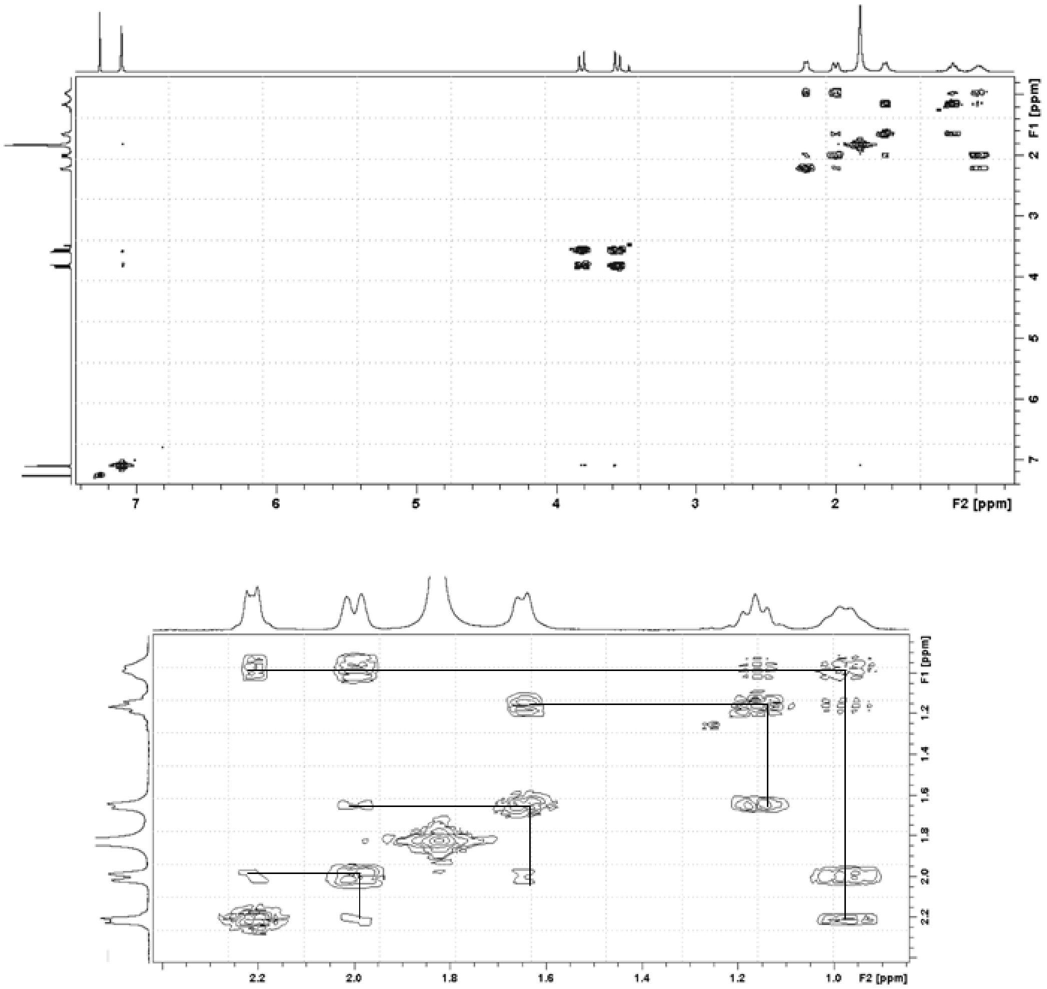

Figure S4. COSY NMR spectra of redCC3 and expanded aliphatic region showing coupling is consistent with assignment.

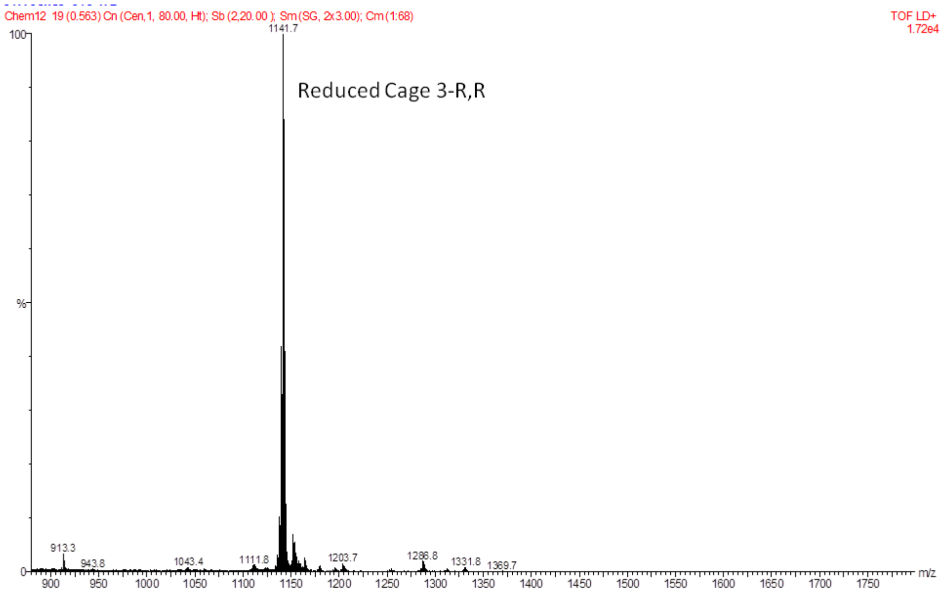

Figure S5. MALDI-TOF mass spectrum of redCC3.

3. Preparation of mixed matrix membranes

MMMs of PIM-1 with CC3 and redCC3 (weight ratios polymer:cage = 10:1, 10:2 and 10:3) were prepared as follows (described for wt. ratio 10:1): PIM-1 (0.22 g) and CC3 (0.022 g) were both separately dissolved in $CHCl_3$ overnight (6 mL and 5 mL respectively). The PIM-1 solution was then filtered through glass wool into the CC3 solution, and the resulting solution stirred overnight. The mixture was then sonicated for 20 min before being poured into a mould. The mould was made up of an 8 cm Teflon ring with a cellophane base. The membrane was allowed to form by slow, solvent evaporation for 3-4 days in a desiccator. The membranes were then kept under N_2 before being transferred to the Institute on Membrane Technology, ITM-CNR, Italy.

MMMs of PIM-1 with nanoCC3 (weight ratios polymer:cage = 10:2 and 10:3) were prepared as follows: PIM-1 was dissolved in DCM (5 mL) overnight. A suspension of nanoCC3 in DCM was sonicated for 15 min. PIM-1 solution was then filtered through glass wool into the desired amount of nanoCC3 suspension. The solution was then stirred overnight, sonicated for 15 min and cast into a Teflon ring with a cellophane base. The membranes were then left to dry for 7 days or until the membranes had lifted off the cellophane.

4. Visual appearance of mixed matrix membranes

Photographs of representative MMMs are shown in **Figure S6**. PIM-1/CC3 membranes were opaque, due to the presence of relatively large crystals, whereas redCC3 and nanoCC3 membranes were transparent. A mottled effect in PIM-1/nanoCC3 membranes may be attributed to fast evaporation of the volatile casting solvent (DCM) and to the formation of occasional clusters of nanoparticles.

a) **b)** **c)**
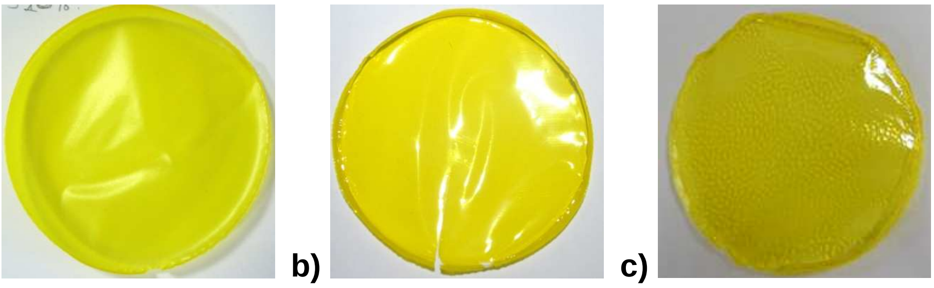

Figure S6. Photographs of mixed matrix membranes: (**a**) PIM-1/CC3, (**b**) PIM-1/redCC3, **(c)** PIM-1/nanoCC3, all at wt. ratio 10:2.

5. Gas sorption analysis

Surface areas and pore size distributions were measured by nitrogen adsorption and desorption at 77.3 K using a Micromeritics ASAP 2040 volumetric adsorption analyzer. Samples were degassed offline at 100 °C for 15 h under vacuum (10^{-5} bar) before analysis, followed by degassing on the analysis port under vacuum, also at 100 °C. Nitrogen adsorption/desorption isotherms for PIM-1, CC3 and a representative PIM-1/CC3 MMM are shown in **Figure S7**.

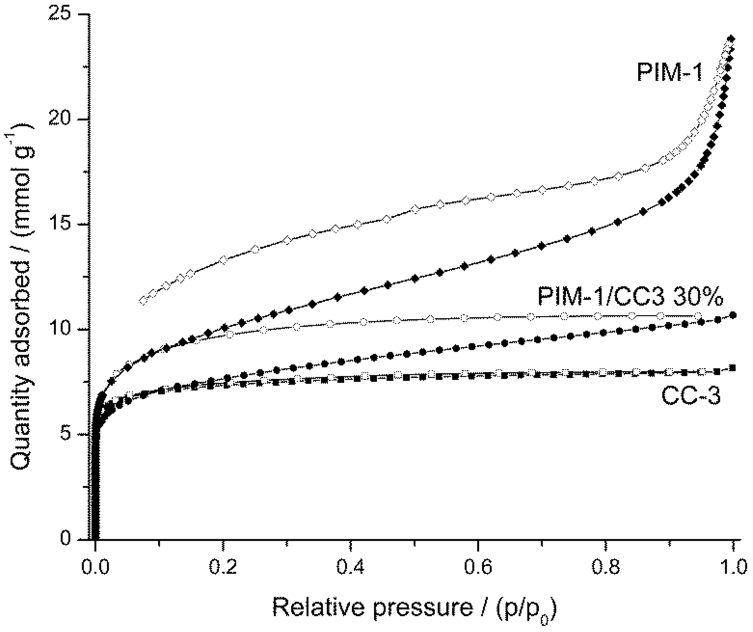

Figure S7. Nitrogen adsorption (filled symbols) and desorption (open symbols) isotherms at 77 K for PIM-1 (◆,◇), CC3 (■,□) and a PIM-1/redCC3 MMM (wt. ratio 10:3) (●,○).

6. Scanning Electron Microscopy (SEM)

The membrane morphology was studied by scanning electron microscopy (SEM) using a FEI Quanta 200 ESEM. The membrane cross section was prepared via freeze fracturing using liquid nitrogen. The sample was then coated with gold via sputtering using an Emitech coater. Representative SEM images are shown in **Figure S8**.

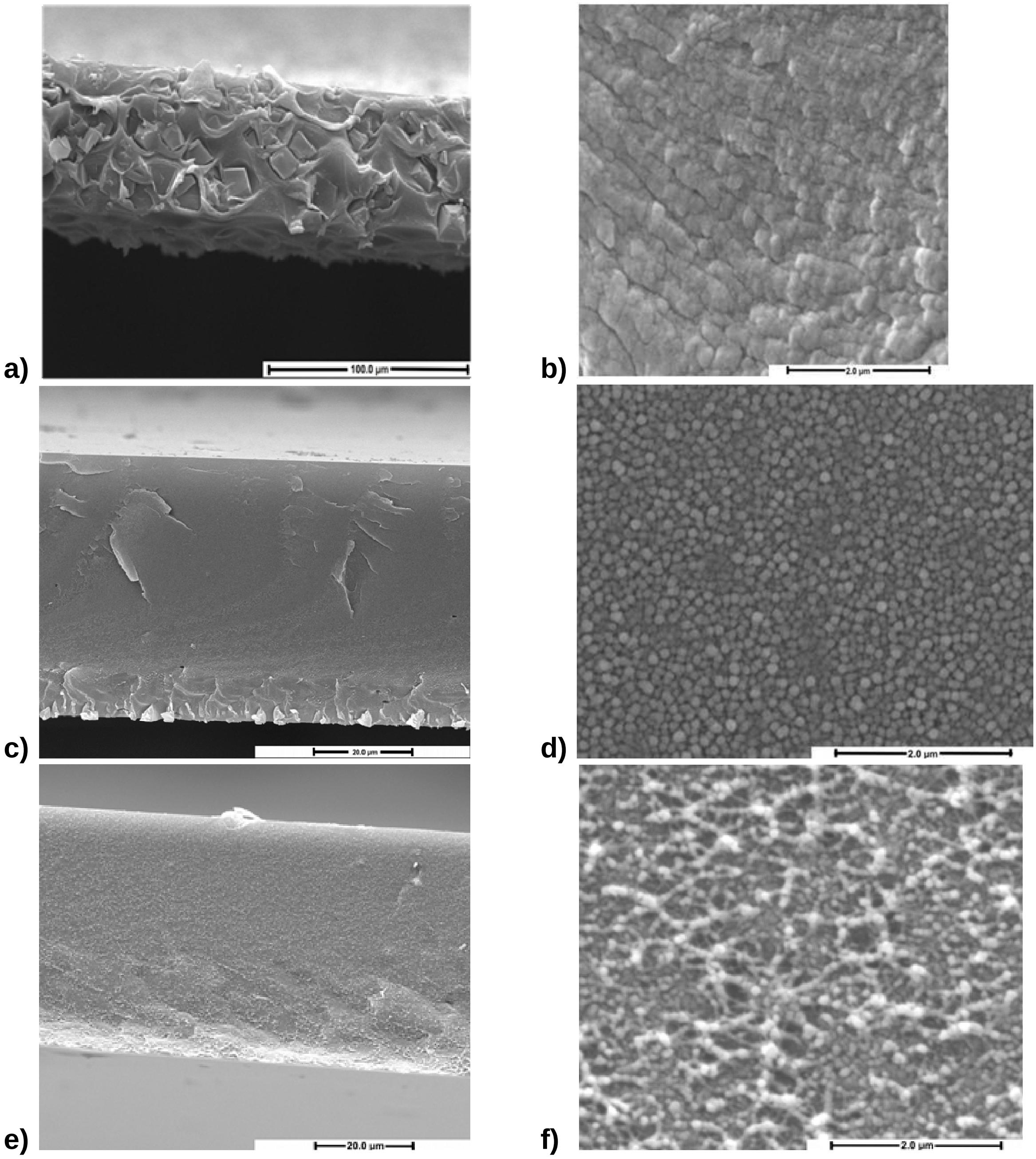

Figure S8. SEM images of cross sections of **(a)** PIM-1/CC3, **(c)** PIM-1/redCC3 and **(e)** PIM-1/nanoCC3, MMMs, and high magnification SEM images of **(b)** PIM-1/CC3 (amorphous polymeric phase), **(d)** PIM-1/redCC3 and **(f)** PIM-1/nanoCC3, all at wt. ratio 10:2. A nanostructure visible in PIM-1/redCC3 membranes **(d)**, and in the continuous, amorphous phase of PIM-1/CC3 membranes **(b)**, may reflect microphase separation during membrane formation.

7. X-ray diffraction (XRD)

X-ray diffraction patterns were collected using a Philips High X'pert Pro Diffraction System Model PW3040/60 and X'Pert Data Collector version 2.0d software, with Cu Kα X-ray radiation. A 5 mm × 5 mm square from the central part of the membrane was placed on a silicon plate. The sample was then scanned from 5-30 degrees in the 2θ range. Representative XRD patterns are shown in **Figure S9**.

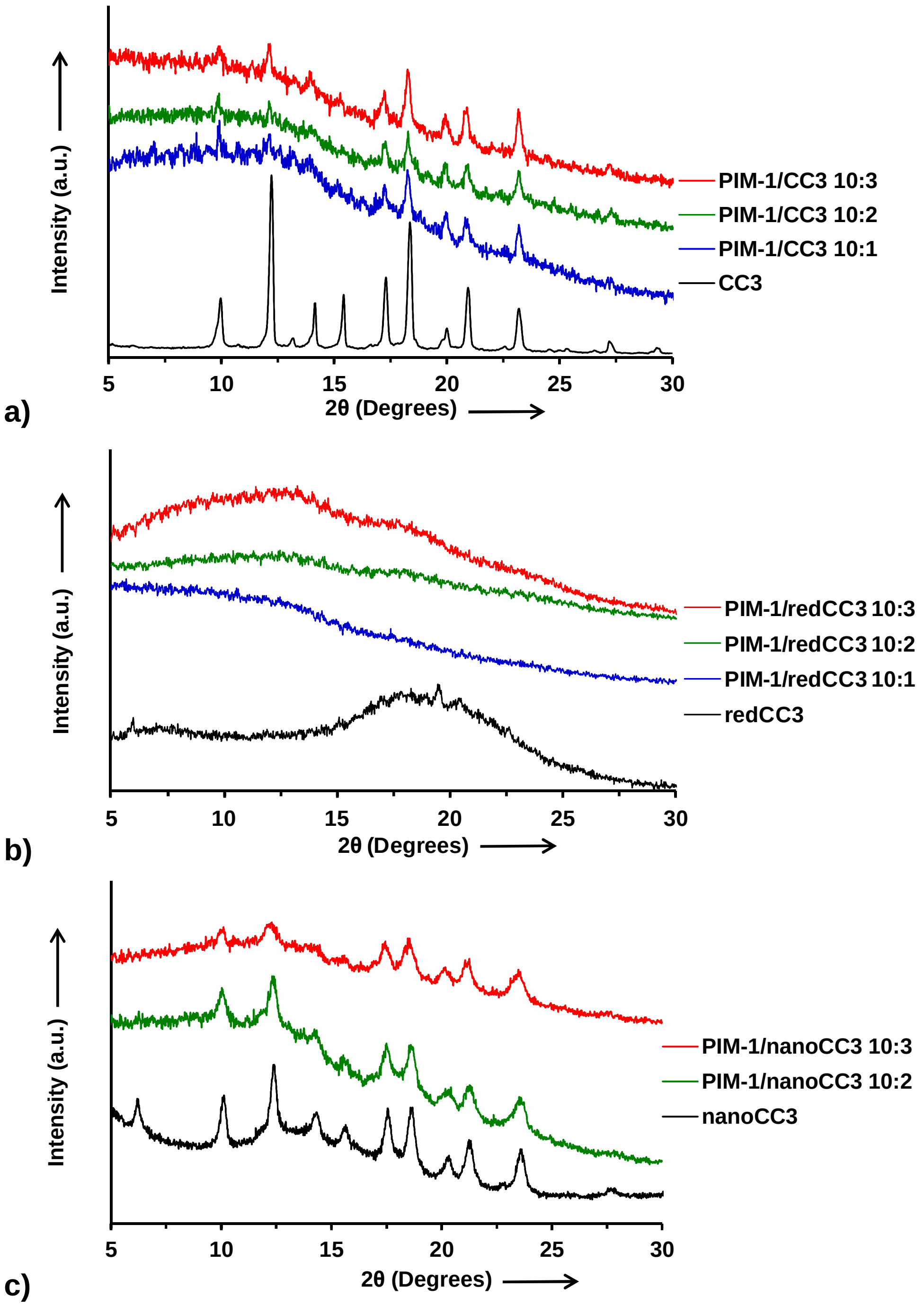

Figure S9. XRD patterns for (**a**) CC3 powder and PIM-1/CC3 MMMs, (**b**) redCC3 powder and PIM-1/redCC3 MMMs, **(c)** nanoCC3 powder and PIM-1/nanoCC3 MMMs.

8. Area powder X-ray diffraction

Area Powder Diffraction patterns were collected using a PANalytical X'Pro MPD diffractometer using Cu Kα radiation. The membrane samples were mounted on a XYZ high throughput stage and data recorded in transmission geometry (**Figure S10**). The incident beam was conditioned using a Cu focusing mirror with a beam mask of 4 mm and 0.04 rad soller slits. The diffracted beam was processed using a linear PIXcel detector operating in scanning mode. Data were collected from 4 - 50 2θ. Representative results are shown in **Figure S11**.

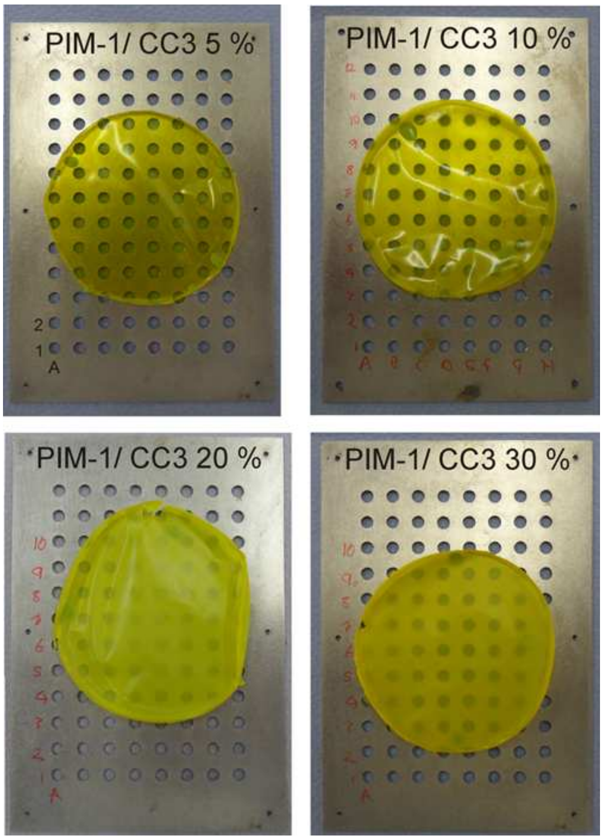

Figure S10. Experimental setup for area powder XRD experiments of PIM-1/CC3 MMMs, where each circular hole represents an individual data collection.

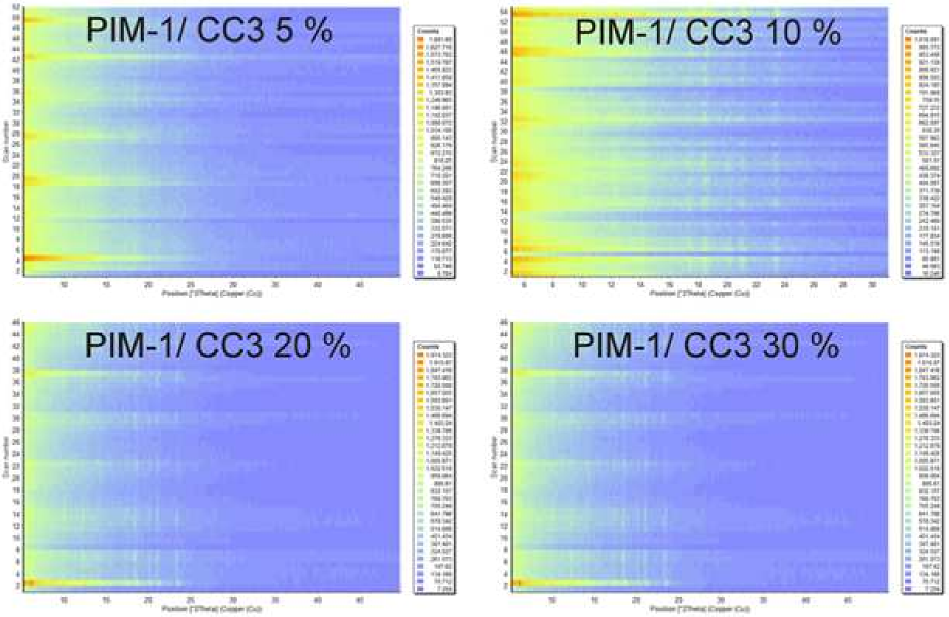

Figure S11. Area Powder XRD patterns of PIM-1/CC3 MMMs illustrating relative uniformity of CC3 distribution across the membrane.

9. Single gas permeation measurements

Gas permeation tests of single gases were carried out at 25 °C and at a feed pressure of 1 bar, using a fixed-volume pressure increase instrument.

Before analysis the membrane samples were carefully evacuated to remove previously dissolved species. The gases were tested in the following order: He, N_2, O_2, CH_4, CO_2. A total membrane area of 2.14 cm^2 was used. The pressure increase in the permeate volume was monitored by a pressure transducer, starting from the instant of exposure of the membrane to the feed gas. Permeability coefficient, *P*, was calculated from the slope of the pressure-time curve in the steady state condition:

$$p_t = p_0 + \left(dp/dt\right)_0 \cdot t + \frac{RT \cdot A}{V_P \cdot V_m} \cdot \frac{p_f \cdot P}{l}\left(t - \frac{l^2}{6D}\right) \quad (1)$$

in which p_t is the permeate pressure at time *t*, p_0 the starting pressure, $(dp/dt)_0$ the baseline slope, p_f the feed pressure, *R* the universal gas constant, *T* the absolute temperature, *A* the exposed membrane area, V_P the permeate volume, V_m the molar volume of the permeating gas at standard temperature and pressure (0 °C and 1 atm). The term $p_0 + (dp/dt)_0$ accounts for the starting pressure and the baseline slope and is normally negligible in the case of well evacuated defect free samples.

The time lag method was applied to determine the diffusion coefficient, *D*, of each gas through the membranes, according to the equation

$$D = \frac{l^2}{6\theta} \quad (2)$$

where *l* is the membrane thickness and θ is the gas time lag. Assuming the validity of the solution-diffusion transport mechanism, the solubility coefficient, *S*, was obtained indirectly via the equation:

$$S = P / D \quad (3)$$

Separation of gas pairs in polymeric membranes is defined by the permselectivity, obtained as the ratio of the permeability for two gases:

$$\alpha_{A/B} = P_A/P_B \quad (4)$$

Selectivity can be decoupled into solubility-selectivity and diffusivity-selectivity

$$\alpha_{A/B} = S_A/S_B \times D_A/D_B \quad (5)$$

Five thickness measurements were made for each membrane sample with a digital micrometer (Mitutoyo).

Single gas permeation results for PIM-1/CC3, PIM-1/redCC3 and PIM-1/nanoCC3 MMMs are tabulated in **Tables S1, S2 and S3**, respectively. Results for as prepared membranes are plotted in **Figure S12** and for ethanol-treated membranes in **Figure S13**. "Robeson" double logarithmic plots of selectivity against permeability are shown in **Figure S14** for the CO_2/N_2, CO_2/CH_4 and O_2/N_2 gas pairs.

Table S1: Single gas permeation data for PIM-1/CC3 MMMs. *P* is permeability coefficient (Barrer*), *D* is diffusion coefficient (10^{-12} m^2 s^{-1}), *S* is solubility coefficient (cm^3[STP] cm^{-3} bar^{-1}), α is ideal selectivity relative to N_2 (P_i/P_{N2}).

History	weight ratio PIM-1:cage, thickness		N_2	CH_4	O_2	He	CO_2
As prepared	10:0 (PIM-1), 51.5±2.4 µm	*P*	120	170	510	800	2790
		D	36	12	124	**	46
		S	2.6	11	3.1	**	45
		α		1.4	4.4	6.6	23
	10:1, 53.3±1.2 µm	*P*	150	230	560	740	3250
		D	42	15	142	**	61
		S	2.6	11	2.9	**	40
		α		1.5	3.8	5.0	22
	10:2, 62.3±2.3 µm	*P*	220	380	770	920	4870
		D	81	28	250	**	120
		S	2.1	10	2.3	**	31
		α		1.7	3.5	4.1	22
	10:3, 67±1.5 µm	*P*	270	480	820	820	5430
		D	95	34	250	**	130
		S	2.1	11	2.4	**	32
		α		1.8	3.0	3.1	20
Ethanol treated	10:0 (PIM-1), 64.3±1.6 µm	*P*	660	1000	1950	1760	11400
		D	145	50	390	**	170
		S	3.4	14	3.8	**	50
		α		1.5	3.0	2.7	17
	10:1, 59.6±2.3 µm	*P*	1350	2050†	3150	2310	18780
		D	310	120†	640	**	300
		S	3.3	13†	3.7	**	47
		α		1.7†	2.3	1.7	14
	10:2, 68.4±1.1 µm	*P*	2360	4860	4970	3470	27100
		D	610	310	1120	**	500
		S	2.9	12	3.3	**	40
		α		2.1	2.1	1.5	11
	10:3, 85.1±5.4 µm	*P*	3270	7220	6810	4380	37400
		D	950	510	1690	**	780
		S	2.6	11	3.0	**	36
		α		2.2	2.1	1.3	11

*1 Barrer = 10^{-10} cm^3[STP] cm cm^{-2} s^{-1} $cmHg^{-1}$ = 3.35×10^{-16} mol m m^{-2} s^{-1} Pa^{-1}

**For He the time-lag was too short for an accurate determination of *D*, and hence *S*.

†Methane data were not obtained for the original PIM-1:CC3 10:1 ethanol-treated MMM, so values are given for a fresh sample, of thickness 52.2 µm (values for other gases are similar to those quoted here).

Table S2: Single gas permeation data for PIM-1/redCC3 MMMs. *P* is permeability coefficient (Barrer*), *D* is diffusion coefficient (10^{-12} m^2 s^{-1}), *S* is solubility coefficient (cm^3[STP] cm^{-3} bar^{-1}), α is ideal selectivity relative to N_2 (P_i/P_{N2}).

History	weight ratio PIM-1:cage, thickness		N_2	CH_4	O_2	He	CO_2
As prepared	10:0(PIM-1), 51.5±2.4 μm	*P*	120	170	510	800	2790
		D	36	12	124	**	46
		S	2.6	11	3.1	**	45
		α		1.4	4.4	6.6	23
	10:1, 27.9±0.90 μm	*P*	60	90	240	370	1480
		D	20	6.6	65	**	28
		S	2.4	10	2.7	**	40
		α		1.5	3.8	5.9	24
	10:2, 49.8±0.75 μm	*P*	30	50	120	230	780
		D	14	4.8	46	**	19
		S	1.8	7.7	2.0	**	31
		α		1.5	3.7	7.1	24
Ethanol treated	10:0 (PIM-1), 64.3±1.6 μm	*P*	660	1000	1950	1760	11400
		D	145	50	390	**	170
		S	3.4	14	3.8	**	50
		α		1.5	3.0	2.7	17
	10:1, 34.4±1.4 μm	*P*	280	400	925	1090	5510
		D	59	20	180	**	77
		S	3.5	15	3.9	**	53
		α		1.4	3.4	3.9	20
	10:2, 58.9±1.2 μm	*P*	190	282	616	808	3720
		D	57	19	180	**	73
		S	2.5	11	2.6	**	38
		α		1.5	3.2	4.3	20

*1 Barrer = 10^{-10} cm^3[STP] cm cm^{-2} s^{-1} $cmHg^{-1}$ = 3.35×10^{-16} mol m m^{-2} s^{-1} Pa^{-1}
**For He the time-lag was too short for an accurate determination of *D*, and hence *S*.

Table S3: Single gas permeation data for PIM-1/nanoCC3 MMMs. *P* is permeability coefficient (Barrer*), *D* is diffusion coefficient (10^{-12} m^2 s^{-1}), *S* is solubility coefficient (cm^3[STP] cm^{-3} bar^{-1}), α is ideal selectivity relative to N_2 (P_i/P_{N2}).

History	weight ratio PIM-1:cage, thickness		N_2	CH_4	O_2	He	CO_2
As prepared	10:0 (PIM-1), 51.5±2.4 µm	*P*	120	170	510	800	2790
		D	36	12	120	**	46
		S	2.6	11	3.1	**	45
		α		1.4	4.4	6.6	23
	10:2, 27.9±0.90 µm	*P*	540	790	1690	1330	9730
		D	140	47	390	**	180
		S	2.8	13	3.3	**	41
		α		1.5	3.1	2.5	18
	10:3, 49.8±0.75 µm	*P*	350	620	980	745	5890
		D	100	36	260	**	130
		S	2.7	13	2.9	**	35
		α		1.7	2.8	2.1	17
Ethanol treated	10:0 (PIM-1), 64.3±1.6 µm	*P*	660	1000	1950	1760	11400
		D	145	50	390	**	170
		S	3.4	14	3.8	**	50
		α		1.5	3.0	2.7	17
	10:2, 34.4±1.4 µm	*P*	770	995	2910	2720	14920
		D	210	66	580	**	240
		S	2.8	11	3.8	**	47
		α		1.3	3.8	3.5	19
	10:3, 58.9±1.2 µm	*P*	1010	1280	3720	3010	18090
		D	200	55	620	**	260
		S	3.8	17	4.5	**	53
		α		1.3	3.7	3.0	18

*1 Barrer = 10^{-10} cm^3[STP] cm cm^{-2} s^{-1} $cmHg^{-1}$ = 3.35×10^{-16} mol m m^{-2} s^{-1} Pa^{-1}
**For He the time-lag was too short for an accurate determination of *D*, and hence *S*.

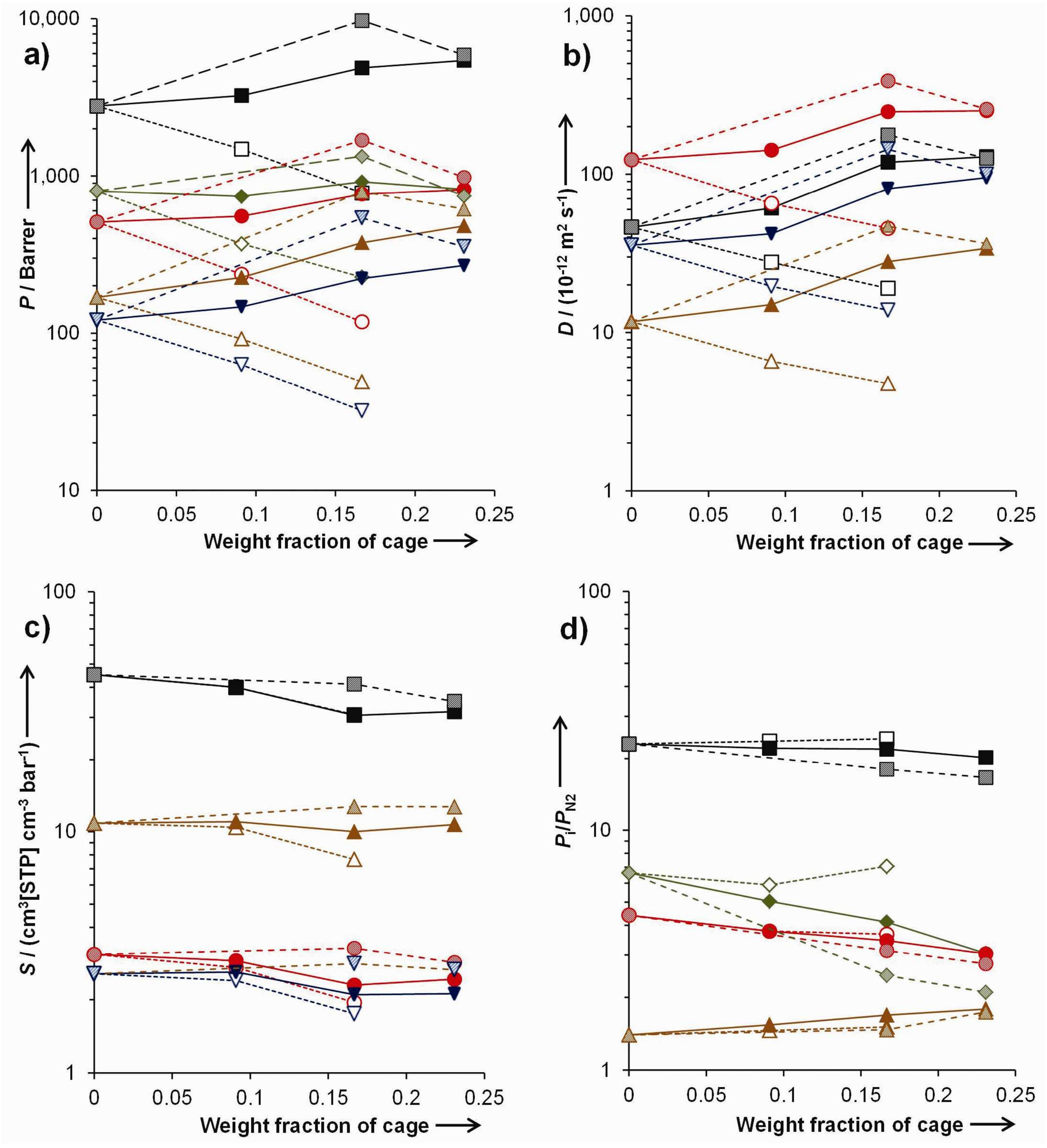

Figure S12. Dependence of **(a)** permeability coefficient, **(b)** diffusion coefficient, **(c)** solubility coefficient and **(d)** ideal selectivity relative to N_2, for CO_2 (■), He (◆), O_2 (●), CH_4 (▲) and N_2 (▼), on weight fraction of cage for as-prepared PIM-1/CC3 (filled symbols, solid lines), PIM-1/redCC3 (open symbols, short dashes) and PIM-1/nanoCC3 (shaded symbols, long dashes).
Unit of permeability: 1 Barrer = 10^{-10} cm^3[STP] cm cm^{-2} s^{-1} cmHg^{-1} = 3.35×10^{-16} mol m m^{-2} s^{-1} Pa^{-1}.
For He the time-lag was too short for an accurate determination of *D*, and hence *S*.

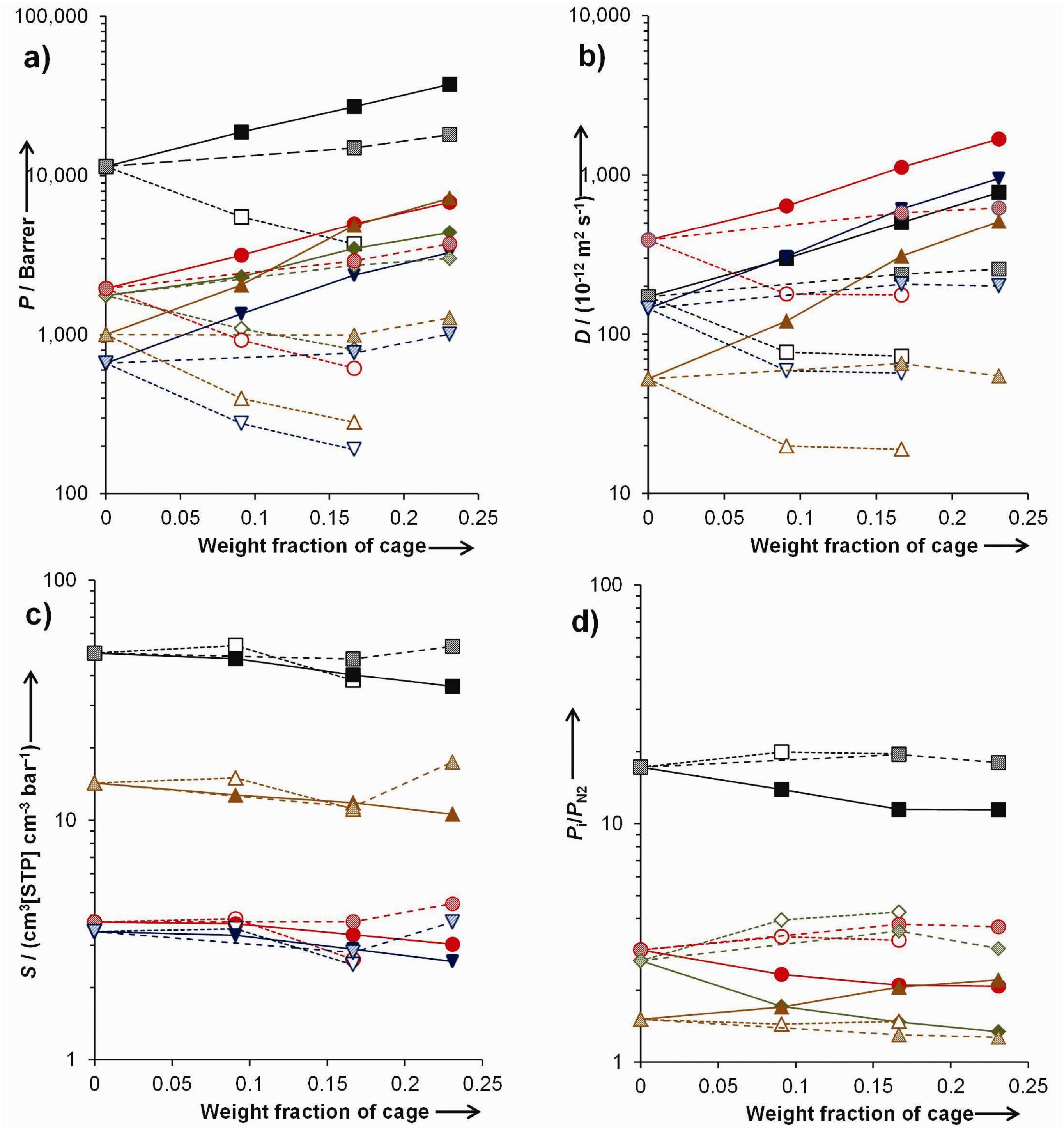

Figure S13. Dependence of **(a)** permeability coefficient, **(b)** diffusion coefficient, **(c)** solubility coefficient and **(d)** ideal selectivity relative to N_2, for CO_2 (■), He (◆), O_2 (●), CH_4 (▲) and N_2 (▼), on weight fraction of cage for ethanol-treated PIM-1/CC3 (filled symbols, solid lines), PIM-1/redCC3 (open symbols, short dashes) and PIM-1/nanoCC3 (shaded symbols, long dashes).

Unit of permeability: 1 Barrer = 10^{-10} cm^3[STP] cm cm^{-2} s^{-1} $cmHg^{-1}$ = 3.35×10^{-16} mol m m^{-2} s^{-1} Pa^{-1}.

For He the time-lag was too short for an accurate determination of *D*, and hence *S*.

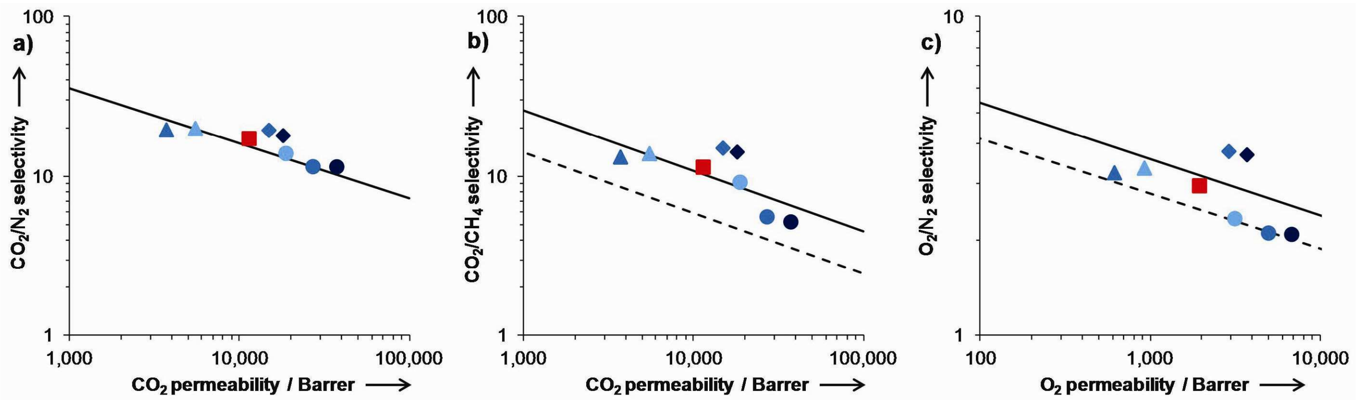

Figure S14. Relationship between **(a)** CO_2/N_2 selectivity and CO_2 permeability, **(b)** CO_2/CH_4 selectivity and CO_2 permeability, and **(c)** O_2/N_2 selectivity and O_2 permeability, showing Robeson's 1991 (- - -) and 2008 (——) upper bounds,[7] with data for freshly ethanol-treated PIM-1 (■), and for PIM-1/CC3 MMMs (●,●,●), PIM-1/redCC3 MMMs (▲,▲) and PIM-1/nanoCC3 MMMs (◆,◆) at weight ratios 10:1 (●,▲), 10:2 (●,▲,◆) and 10:3(●,◆).

10. Mixed gas permeation measurements

Mixed gas permeation tests were carried out on a PIM-1/CC3 (wt. ratio 10:3)) sample aged for 258 days, using a synthetic ternary mixture (molar ratio CO_2:O_2:N_2 = 35:10:55, Pirossigeno, Italy) which simulates the composition of typical flue gases from steel production or from lime kilns.

Experiments were carried out at ambient temperature and at a total feed pressure up to 6.5 bar. The effective membrane area was 2.14 cm^2. Argon sweep gas was introduced at a flow rate of 30 cm^3(STP) min^{-1} into the permeate side of the cell by means of a thermal mass flow controller (Bronkhorst Hi-Tech). The feed pressure was controlled by a forward pressure controller (Swagelok) on the feed stream, while the retentate flow rate was controlled at a value of at least 30 times the total permeate flow rate in order to keep a low stage cut (permeate to feed flow rate ratio). The permeate was at ambient pressure and the stage-cut for all the measurements was less than 2.5%. Under these conditions, the residue composition was essentially equal to that of the feed and concentration polarization effects were negligible. Permeability of pure CO_2 and N_2 was determined under identical conditions for comparison, also using argon as the sweep gas.

The compositions of feed, residue, and permeate were determined with a mass spectrometer (Hiden Analytical, HPR-20 QIC) equipped with a quadrupole mass filter. The electron ionisation energy was 70 eV. A Faraday cup was used as the ion detector. For the calibration procedure, the relative sensitivity of CO_2 in argon and that of N_2 in argon were determined for five or more binary mixtures, prepared online with electronic mass flow controllers. The relative sensitivity of oxygen was determined by calibration of the ternary mixture (CO_2, N_2, O_2) diluted in argon. The nitrogen concentration was determined by appropriate deconvolution of the overlapping signals of N_2 and the CO fragment at 28 AMU.

The mixed CO_2/N_2 selectivity was evaluated as the ratio of the individually calculated gas permeabilities in the mixture:

$$\alpha\left(CO_2/N_2\right) = \frac{P_{CO_2}}{P_{N_2}}$$

where the individual gas permeability of each species, *i*, in the ternary mixture is obtained as the ratio of its volumetric permeate flux, $Q^{Permeate}$, to the partial pressure difference between the feed and permeate sides, Δp_i:

$$P_i = \frac{x_i^{Permeate} Q^{Permeate}}{\Delta p_i} l = \frac{x_i^{Permeate} Q^{Permeate}}{x_i^{Feed} p^{Feed} - x_i^{Permeate} p^{Permeate}} l$$

in which x_i is the mole fraction of the ith species, p^{Feed} and $p^{Permeate}$ are the total feed and permeate pressures, respectively and l is the membrane thickness. The permeate flux is the flow rate per unit area, defined as:

$$Q^{Permeate} = \frac{J^{Permeate}}{A}$$

The volumetric permeate flow rate, $J^{Permeate}$, is calculated from the known sweep flow rate and from the measured composition of the permeate/sweep mixture.
